# Markov modeling in R: Advanced method using a cost-effectiveness analysis

**DOI:** 10.1371/journal.pone.0350698

**Published:** 2026-06-16

**Authors:** Jean Martial Kouame, Christian Kouakou, Soualio Gnanou, Bilé Yacouba, Carole Siani

**Affiliations:** 1 Département de médecine sociale et préventive, Faculté de médecine, Université Laval, Québec, Canada; 2 Axe Santé Des Populations et Pratiques Optimales en Santé, Centre de Recherche du CHU de Québec Université Laval, Québec, Canada; 3 Université de Sherbrooke, Centre de Recherche de l’Institut Universitaire en Santé Mentale de Montréal, Montreal, Canada; 4 Departement of Operations and Decision Systems, Faculty of Administrative Sciences, Université Laval, Québec, Canada; 5 VITAM-Center for Sustainable Health Research – Université Laval, Quebec, Canada; 6 Research Center of the CHU de Québec – Université Laval, Quebec, Canada; 7 Faculté de Pharmacie, Aix Marseille University, INSERM, IRD, SESSTIM UMR 1252, ISSPAM, Marseille, France; The University of Sydney, AUSTRALIA

## Abstract

A Markov model is the kind of state transition model most widely used in Health Economic Evaluation (HEE) to analyse the efficiency of an intervention and support decision-making. However, it is has transition probabilities which remain constant over time, which limits its use for chronic diseases. Thus, to allow transition probabilities, rewards, or both, to vary over time, we use two types of methods: The first is “Implementing Time Dependency into Markov Transition Probabilities”, which allows probabilities to vary over time as measured from the start of the simulation. The second is “Relaxing the Markov Assumption”, by adding additional health states to the model, called tunnel states. In this tutorial, a case study on breast cancer is used to illustrate how to implement time dependence in a Markov model and how to conduct analyses of cost-effectiveness, probabilistic sensitivity, and the value of perfect information analyses with R.

## Introduction

The foundation of many health economic evaluations is often a cohort state-transition model, commonly known as a Markov model. Such models are generally used to evaluate a health system, such as health technologies, diagnostic tests, and medical interventions [1–6].

The objective of heath economic evaluations is to help decision-makers make an efficient 1allocation of resources, by determining, between several considered strategies, which is optimal on the two-fold basis of its cost and its effectiveness (including the patients’ quality of life). Thus, the analysis for the decision-making needs to adequately reflect the key features of the natural history of the disease and the impact of alternative programmes and interventions [7].

Although the simplest Markov model provides greater flexibility than a decision tree, it also has some important restrictions in the context of structuring complex prognoses, due to the Markov assumption, or the fact that Markov models are memoryless. This assumption means once a patient has moved from one state to another the Markov model will have “no memory’‘ of where the patient has come from or the timing of that transition [8]. In addition, simple Markov models are often not adapted to chronic diseases, because the cost, utility scores (corresponding to an estimate of the patient’s quality of life) and transition probabilities may change over time. For example, mortality can change as the population ages, the risk of a recurrence of a cancer might vary as a function of the time since diagnosis [7].

Thus, for certain types of decision-making problems, it is important to go beyond the simple Markov model, by combining a decision tree with the cohort model; or adding time-dependency; or relaxing the memoryless restriction of the Markov model, which might improve its adequacy for the disease or the relevant interventions. Because the costs and utility scores are associated with the health of the patient, the state might also vary over time [7]. For instance, the transition probability, costs and utility scores related to cancer may change over time, depending on whether a cohort is in their first year of remission or in their third. Therefore, models including time-dependency must capture this variation. The main justification of this tutorial nowadays is that, if extensive time-dependency is required, however, a large number of tunnel states may be necessary. In such a case, programming the model in a spreadsheet can be very difficult. One way around this is to use a mathematical programming language such as R, which facilitates multidimensional transition probabilities.

**Fig 1:**
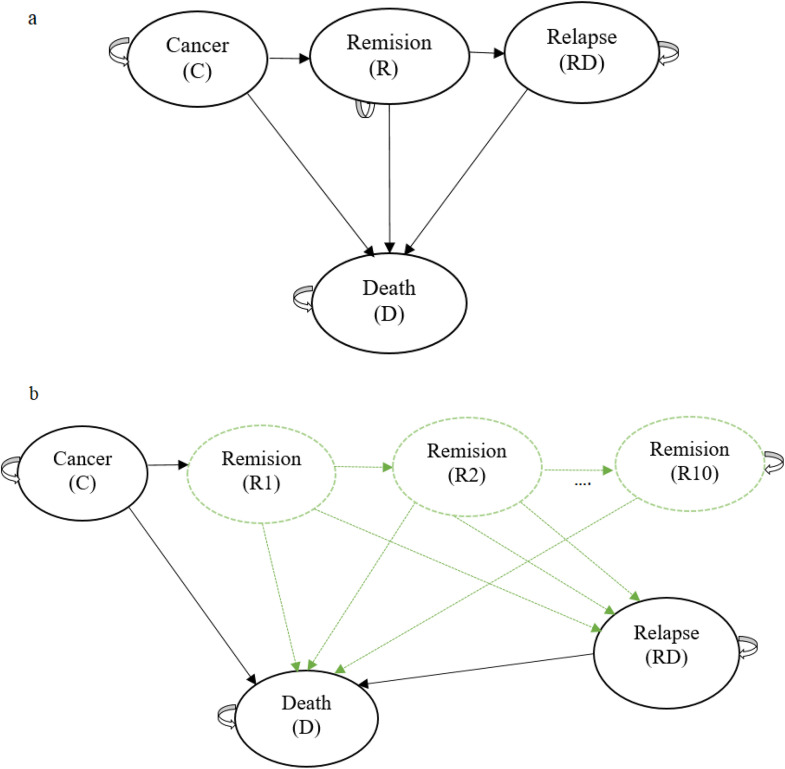
a. First breast cancer model. b. Breast cancer model with R tunnel state (revised model).

Our objective in this tutorial is to illustrate how to include time-dependency and how to relax the Markov assumption in a Markov model. In addition, we describe step-by-step how to carry out a cost-effectiveness analysis (CEA), probabilistic sensitivity analysis (PSA), and expected value of perfect information (EVPI) with R using a cost effectiveness study (Breast Cancer Model). The reader can find two R codes in the supplementary material to develop Markov model for other chronic pathologies (diabetes, cardiopathologie, kidney etc.). The first one, developed in our first tutorial *Modeling in R: A Practical Application* [8], allows implementing the first Breast Cancer Model (Fig 1a), and the second one shows how to develop a Breast Cancer Model with tunnel states (Fig 1b). In addition issues related to time-dependent transition probabilities and the use of tunnel states in Markov models have also been addressed in methodological papers Alarid-Escudero, F. et al. [7].

II Methodological aspects

In general, Markov assumption means that it may not be straightforward to build ‘history’ into this type of model. That is, to model a process where future events depend on past events. This may be possible by adding additional states to the model and incorporating time dependency into transition probabilities. Both of these extension to the Markov model are considered in the next step.

A Simulation time dependency

The simplest form of time-dependency in a Markov model is where the transition probabilities vary according to how long the cohort has been modelled. Indeed, the cohort’s transition probabilities change with each cycle or as the cohort ages. In the simple Model, the probability of dying is assumed fixed whatever the time. In reality, the annual probability of death should increase as the cohort ages. For this, the initial age of the cohort should be known because it’s very important for characterizing the patients who benefit from the intervention, particularly when its impact on mortality is being modelled.

If the starting age of the cohort is known, then so is the age of the cohort at any cycle of the model, whatever the state. To implement age-dependent probabilities, therefore, it is necessary to have a (possibly) distinct transition probability for each cycle. Instead of a constant transition probability to state dead, we have a transition probability for each age [9]. With the rate of mortality of each age extracted from France’s national life table (www.ined.fr/fr), we can compute the probability by this formula: p = 1- exp {-rt}, where p is the transition probability, r is the rate of observation of the event of interest, and t is the time-period of interest.

B State time dependency (tunnel state)

The Markov assumption means that once a patient has moved from one state to another, the Markov model will have no memory of where the patient has come from or the timing of that transition. Therefore, the best way to ‘build memory’ into a Markov model is to add additional states, called tunnel states [9]. The tunnel states allow the model to take into account future transitions, as well as the costs and patients’ utilities*.*

Indeed, tunnel states are way of implementing time-dependency by adding memory to a Markov model: patients stay in a tunnel state for 1 cycle, after which patients move to another state (either another tunnel state, or another state) in the next cycle. Such models have been referred to as semi-Markov processes [10]. If extensive time-dependency is required, however, a large number of tunnel states may be necessary. In such a case, programming the model in a spreadsheet can be very difficult.

One way around this is to use a mathematical programming language such as R, which facilitates multidimensional transition probabilities [11]. Currently, adding a tunnel state is the most widely used method to resolve the memoryless aspect of a Markov model.

III Case study: Breast cancer model
  1. Structure and parameters of the model

In this case study, we used the first breast cancer model initially built from a Markov model that compared two strategies, Standard care (SoC) vs Innovative therapy (IT). The model had four health states defined to represent the evolution of the cancer (see Fig 1a): Cancer (C); Remission (R), Relapse (RD) and the state Death (D), which is the absorbing state from which individuals no longer transit. In Fig 1a, individuals in remission will always have the same probability of relapsing, regardless of the cycle. This is the limited memory problem inherent in Markov models.

However, it would be good if the transition probability from R to RD or D could vary depending on the number of years the patient spent in remission. The best way to give Markov models some memory is to create tunnel states. Instead of a single health state (here: remission), successive intermediate states are created that incorporate a notion of time: one year in remission, two years in remission, three or more years in remission, as shown in Fig 1b. The time spent in remission is taken into consideration by assigning different transition probabilities between the three remission states on the one hand, and relapse or death on the other.

In Table 1, all transition probabilities are constant, except when moving from R to D (denoted by p_R-D), and, implicitly, that of moving from R to RD (denoted by p_R-RD). The variation of (p_R-D) as a function of time (cycle) is shown in the last line of Table 1. A specific value of p_R-D is assigned to each tunnel state or cycle of the model. It is indeed the transition probability from the remission state to the death state P_R-D that varies as a function of time (cycles). However, to ensure that the sum of the transition probabilities from the remission state to a state RD is equal to 1 regardless of the cycle, the transition probability from the remission state to the relapse state p_R-RD is implicitly time-dependent. In the transition matrix, this last probability therefore appears in the form 1- p_R-R - P_R-D. Table 1 presented transition matrix of “Standard of Care (SoC) strategy” including a time-dependent transition probability (p_R-D). All the parameters (costs, utility scores and transition probabilities) of the ‘breast Cancer model’ and the names of the R variables are presented in Table 2.

**Table 1:** Transition matrix for Standard Care (SoC) strategy.

	Cancer (C)	Remission (R)	Relapse (RD)	Death (D)
Cancer (C)	0.45	0.4	0	0.15
Remission (R)	0	0.5	**1-** p_R-R **- p_R-D**	p_R-D*
Relapse (RD)	0	0	0.7	0.3
Death (D)	0	0	0	1
p_R-D1 p_R-D2 p_R-D3 p_R-D4 p_R-D5 p_R-D6 p_R-D7 p_R-D8 p_R-D9 p_R-D10 0.05 0.06 0.06 0.07 0.09 0.11 0.14 0.12 0.12 0. 11				

* At each cycle p_R-D takes a value in the last line of this table

**Table 2:** Parameters of Cancer model using R.

Parameter	R name	Base-case values	Distribution
Number of cycles	nb_c	10 years	–
Names of health states	x_n_s	C, R, RD, D	–
Annual discount rate for costs	t_c	2.5%	–
Annual discount rate for QALYs	t_e	2.5%	–
Standard of care (SoC)			
Probability of C becoming R	p_C-R	0.40	rbeta (0.67, 1)
Probability of C becoming D	p_C-D	0.15	rbeta (3.75, 21.27)
Probability of R becoming D	p_R-D	0.05	rbeta (23.7, 450)
Probability of R staying R	p_R-R	0.50	rbeta (2.63, 2.63)
Probability of RD becoming D	p_RD-D	0.30	rbeta (6, 14)
Cost of being in C	c_C	4000€	rgamma (25.89, 154.4)
Cost of being in R	c_R	1000€	rgamma (11.11, 90)
Cost of being in RD	c_RD	5000€	rgamma (25, 200)
Innovative Therapy (IT)			
Probability of C becoming R	p_C-R	0.50	rbeta (50, 48.22)
Probability of C becoming D	p_C-D	0.10	rbeta (10, 7.37)
Probability of R becoming D	p_R-D	0.05	rbeta (2.5, 471,5)
Probability of R staying R	p_R-R	0.50	rbeta (25, 19.75)
Probability of RD becoming D	p_RD-D	0.30	rbeta (0.75, 19.25)
Cost of being in C	c_C	6000€	rgamma (25, 240)
Cost of being in R	c_R	1000€	rgamma (11.11, 90)
Cost of being in RD	c_RD	5000€	rgamma (25, 200)
Utilities scores (SoC and IT)			
Utility of being in C	u_C	0.4	rbeta (2, 3)
Utility of being in R	u_R	0.7	rbeta (0.22, 0.093)
Utility of being in RD	u_RD	0.35	rbeta (7.61, 14.13)

The next sections include R code snippets of the Breast Cancer Model with tunnel state. All the code used in this tutorial and their exact names can be found at https://github.com/KKJM1/MODELING-IN-R-ADVANCED-METHOD-/tree/main

A synthesis of all R codes plus their values as well as an explanation of the development of the

Cancer model are given below.

***Simulation time dependency***

In the breast cancer model, to implement time dependence we used the transition probabilities from the remission to death: we created a vector x_p_R-D to represent the transition probabilities from R to D for each specific age between 65–75, the probabilities being obtained from life tables.

***##*** *Probability of transition to death at each cycle from the remission state*

x_p_R-D < - c(0.05, 0.06, 0.06, 0.07, 0.09, 0.11, 0.14, 0.12, 0.12, 0.11) % > % as.array ()

1 x_p_R-D_tunnels < - rep (x_p_R-D, each = 1/cycle_length)

*## Probability of relapse from remission state*

The transition probability from R to D (p_R-D) varies as a function of time, as does, implicitly, th probability of moving from R to RD (p_R-RD), because p_R-RD is equal to 1- p_R-R –x_p_R-D.

x_p_R-RD_tunnels < - 1- p_R-R - x_p_R-D_tunnels

***## Create transition probability arrays for strategy SoC***

Using R, we created a 3-dimensional transition probability array, M, to introduce time dependency. Thus, for SoC, we initialize the transition probability matrix M_SoC, with zero as the default value for all transition probabilities.

***#*** *Initialize transition probability array for strategy SoC*

M_SoC < - array (0,

dim = c(nb_states, nb_states, nb_cycles),

dimnames = list (x_n_s, x_n_s, 0:(n_cycles - 1)))

Unlike the time-independent first breast cancer model, in revised breast cancer models with time dependence, the transition matrix is a 3-dimensional array (denoted by M_SoC). We created a vector (denoted by x_p_R-D) to integrate the transition probabilities. The R language allows us to generate the transition probability as a function of the number of transition cycles. For the sum of the probabilities for each cycle to be equal to 1, the probability of staying in each state is equal to 1 minus the sum of the probability vectors to the other states. Finally, we use the matrix of SoC to determine Matrix for Strategy B because the cohort is the same.

***## Matrix for Strategy B as a copy of SoC’s***

M_strB < - M_SoC

***## Check if transition probability matrices are valid***

In the matrix, each entry is between 0 and 1 and each probability row must sum to 1. All verification code is in the supplementary material

***Implementation of an iterative solution of age-dependent Model Cancer under SoC***

For (t in 1:nb_cycles){

M_SoC [t + 1,} <- M_SoC [t,} %*% N_SoC [, t]

}

**Fig 2:**
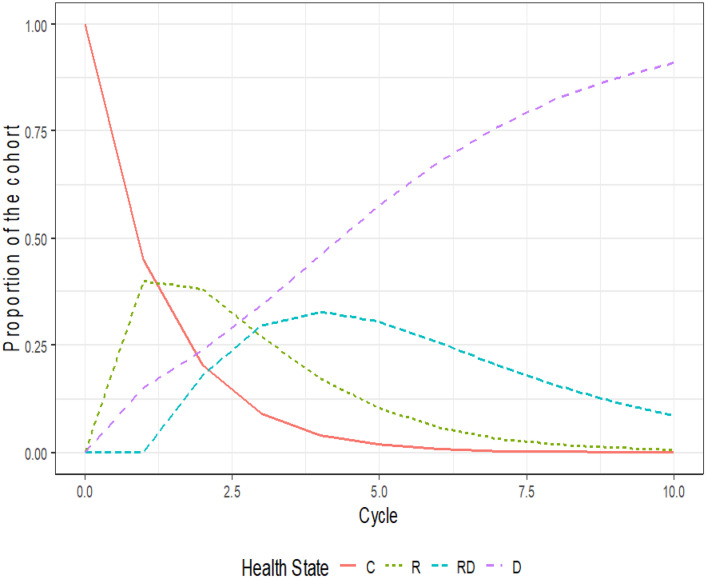
Example of cohort simulation over time under strategy Standard of Care.

The vector of the cohort’s simulation at cycle t + 1 is equal to the matrix product between the row vector M_SoC [t + 1,} and the transition probability matrix N_SoC[, t]. Fig 2 presents simulation over time of the cohort under strategy SoC. Each hypothetical individual starts in the state C and moves to other states over time. For instance, in the strategy SoC, we observe that the proportion of the cohort in state C decreases from one to zero and the proportion in D increases quickly. After that, the proportion of the cohort in R and RD increases to 0.4 and decreases at the end of the time horizon, this could be explained by the little efficacy of SoC.

***State time dependency (tunnel state)***

Adding tunnel states is way of implementing time-dependency in a Markov model: the cohort resides in each transient tunnel state for exactly 1 cycle, then they pass to another state, such as RD or D, or the next tunnel state. In our case study, the progression from R to RD and D depends on how long the patient spent in the R state. Thus, we developed a new variable to represent the number of tunnels (nb_tunnel_size) from the number of cycle (nb_cycles). We have ten tunnel states (kinds of remission states) plus three more states (C, RD, D). In practice, we added a tunnel state in the cancer model. Then for analysis using the R language, we developed the vector tunnel states of Remission state (x_Remission_tunnel). Finally, we added the names of the remission tunnel states’ and the names of all other states of the breast cancer model in the vector (x_names_states_tunnels). Fig 1b shows a diagram of a cancer model with three tunnel states for remission.

*# Number of tunnels*,

nb_tunnel_size < - nb_cycles

*# Vector with cycles for tunnels*

x_cycles_tunnel < - 1:nb_tunnel_size

*# Vector with names for tunnel states of Remission state*

x_ Remission_tunnel < - paste (“R_”, seq (1, nb_tunnel_size), “Yr”, sep = ““)

*# create variables for model with tunnels*

x_names_states_tunnels < - c(“C”, x_ Remission_tunnel, “RD”, “D”) *# health state names*

nb_states_tunnels < - length (x_names_states_tunnels) *# number of health states*

*# Initial state vector*

x_m_init_tunnels < - c(1, rep(0, n_tunnel_size), 0, 0)

***## Create transition probability arrays***

To start we created a 3-dimensional array for the tunnels (N_tunnels_SoC), which will be transformed into a 3-dimensional transition probability matrix to integrate both age and state- residence dependence in the cancer model under SoC.

*### First transition probability array for strategy SoC*

N_tunnels_SoC < - array(0,

dim = c(nb_states_tunnels, x_states_tunnels, n_cycles),

dimnames = list(x_names_states_tunnels,

x_names_states_tunnels,

0:(n_cycles – 1)))

Adding the transition probabilities in the third-dimensional array for tunnels (N_tunnels_SoC) is a little bit the same as for the matrix of the first breast cancer model (M_SoC) described above. However, in the N_tunnel_SoC, we integrated into the tunnel states the transition probabilities corresponding to the disease’s progression.

***### Filling transition probability array of SoC***

## From C

N_tunnels_SoC [“C”, “C”,] <- 1 - p_C-D - p_C-R

N_tunnels_SoC [“C”, x_ Remission_tunnel [1],] <- p_C-R

N_tunnels_SoC [“C”, “D”,] <- p_C-D

## From R

for (i in 1:nb_tunnel_size) {

N_tunnels_SoC [x_ Remission _tunnel[i], “D”,] <- x_p_R-D_tunnels[i]

N_tunnels_SoC [x_ Remission_tunnel[i], “RD”,] <- 1 – p_R-R – x_p_R-D_tunnels[i]

if (i == nb_tunnel_size) {

N_tunnels_SoC [x_ Remission_tunnel[i],

x_Remission_tunnel[i],} <- p_R-R

} else{

N_tunnels_SoC [x_ Remission _tunnel[i],

x_Remission_tunnel[i + 1],} <- p_R-R

}

}

## From RD

N_tunnels_SoC [“RD”, “RD”,] <- 1 - p_RD-D

N_tunnels_SoC [“RD”, “D”,] <- p_RD-D

## From D

N_tunnels_SoC [“D”, “D”,] <- 1

***### Initialize cohort trace for state-residence under SoC***

M_tunnels_SoC < - matrix (0,

nrow = (n_cycles + 1), ncol = x_states_tunnels,

dimnames = list (0:n_cycles, x_names_states_tunnels))

*## Add the first state vector into the matrix*

M_tunnels_SoC [1,] <- x_m_init_tunnels

To obtain the complete cohort trace, we carry out the matrix product between the row vector M_tunnels_SoC [t,] and the transition probability matrix N_tunnels_SoC[, t].

*## Iterative solution of state-residence dependent*

for (t in 1:n_cycles) {

M_tunnels_SoC[t + 1,} <- M_tunnels_SoC[t,} %*% N_tunnels_SoC[, t]

}

To calculate the cohort trace for each of the states C, R, RD, and D under SoC, we aggregate over the R tunnel states in each cycle.

*## Create aggregated trace*

M_tunnels_SoC_sum <- cbind (C = M_tunnels_SoC[, “C”],

R = rowSums(M_tunnels_SoC [, 2:(nb_tunnel_size + 1)]),

RD = M_tunnels_SoC [, “RD”],

D = M_tunnels_SoC [, “D”])

IV Cost and effectiveness outcomes
  1. State rewards

State rewards (**Q)** is equal to T*R. To estimate **Q**, we multiply the transition-probabilities array (**T**) by the Arrays of state and transition rewards (**R**).


$$\begin{array}{c} 𝐐\;=\;𝐓*𝐑. \end{array}$$ (1)

The result of equation (1) provides the estimated costs and QALYs for both the strategies. Before computing Q, we describe below how to develop, transition- probabilities array (**T**) and the Arrays of state and transition rewards (**R**).

***## Initialize transition dynamics arrays, which will capture transitions***

**T**_SoC < - array (0,

dim = c(nb_states, nb_states, nb_cycles + 1),

dimnames = list(x_n_s, x_n_s, 0:nb_cycles))

*## Add the initial state vector in the diagonal of the array*

diag (T_SoC[, 1]) <- x_m_init

After that, we multiply each row of the diagonal matrix under SoC (diag(M_SoC[t,})) by the array of the transition matrix (N_SoC[, t]) over all the cycles. We use an iterative method to generate the transition-dynamics array under SoC.

for (t in 1:n_cycles){

**T**_SoC [, t + 1] <- diag(M _SoC[t,}) %*% N_SoC[, t]

}

***## Array of state and transition rewards***

*# create transition matrices of utility under SoC*

m_u_str < - matrix (x_u_SoC, nrow = n_states, ncol = n_states, byrow = T)

**R**_u_ SoC < - array (m_u_SoC,

dim = c(nb_states, nb_states, n_cycles + 1),

dimnames = list (x_names_states, x_names_states, 0:n_cycles))

*# create transition matrices of cost under SoC*

m_c_SoC < - matrix (x_c_SoC, nrow = n_states, ncol = n_states, byrow = T)

**R**_c_SoC < - array (m_c_SoC,

dim = c(nb_states, nb_states, n_cycles + 1),

dimnames = list (x_names_states, x_names_states, 0:n_cycles))

To get rewards (**Q**) for costs and QALYs for both the strategies, we multiply of the transition array T_SoC by the array of rewards R_x_SoC, where x can be either cost or QALYs, (see formulae below).

**Q**_c_SoC < - **T**_SoC * **R**_c_SoC

**Q**_u__SoC < - **T**_SoC * **R**_u_SoC

***## Expected QALYs and costs per cycle***

x_qaly_SoC < - apply (**Q**_u_SoC, 3, sum) *# sum the proportions of the cohort across transitions*

x_cost_SoC < - apply (**Q**_c_SoC, 3, sum) *# sum the proportions of the cohort across transitions*

1. Within-cycle correction and discounting

We applied Simpson’s one-third rule for within-cycle correction, [12,13] and exponential discounting for costs and QALYs. For the code for these within-cycle corrections and discounting, see the Supplementary Material

***## Discounted total expected QALYs and Costs per strategy and apply within-cycle correction***

*# Total QALYs*

x_tot_qaly < - t(x_qaly_SoC) %*% (x_dwe * x_wcc)

*# Total Costs*

x_tot_cost < - t(x_cost_SoC) %*% (x_dwc * x_wcc)

V Incremental cost-effectiveness ratios (ICERs)

To estimate the incremental cost-effectiveness ratio (ICER), a commonly used decision-making indicator, we used the total expected discounted costs and QALYs calculated before and we took the difference between the average costs and then between average QALYs of both strategies. The results have been stored into “c_incr’‘ for the incremental costs and “q_incr’‘ for the incremental QALYs. After that, ICER is equal to the ratio of the incremental costs and incremental average QALYs of both strategies (ratio of “c_incr’‘ and “q_incr’‘).

***### Calculate incremental cost-effectiveness ratios (ICERs)***

c_incr < - nb_tot_cost_strB- nb_tot_cost_SoC *# Creation of object to store incremental cost*

q_incr < - nb_tot_qaly_strB - nb_tot_qaly_SoC *# Creation of object to store incremental qalys*

***### Incremental cost-effectiveness ratio***

ICER < - c_incr/ q_incr *# Creation of a vector to store the ICER*

The Innovative therapy (B) is more costly and effective than Standard of Care (SoC), with an effectiveness of 1.88 QALYs, cost of € 16354.72. In addition, strategy B is efficient because

The ICER of €16065.39/ QALY is less than the willingness to pay (€ 30000). All results are presented in Table 3.

**Table 3:** Cost-Effectiveness Analysis.

	Cost (€)	QALYs	c_incr	q_incr	ICER (€/QALY)
Standard of care	13601.93	1.88	-	-	-
Innovative therapy	16354.72	2.05	2752.78	0.17	16065.39

VI Probabilistic sensitivity analysis

To handle the uncertainty of the hypotheses made about the model parameters (transition probability, cost, utility scores) and their impact on the ICER, we carried out a probabilistic sensitivity analysis (PSA) [14]. In practice, we applied Beta distributions to the transition probabilities and utility scores and Gamma distributions to costs. Finally, we calculated the ICER with the new sample of model parameter values. The PSA method has been described in our previously published article and all R code can be found in the supplementary material. We share with you a step-by-step method, which is the easiest method.

**Fig 3:**
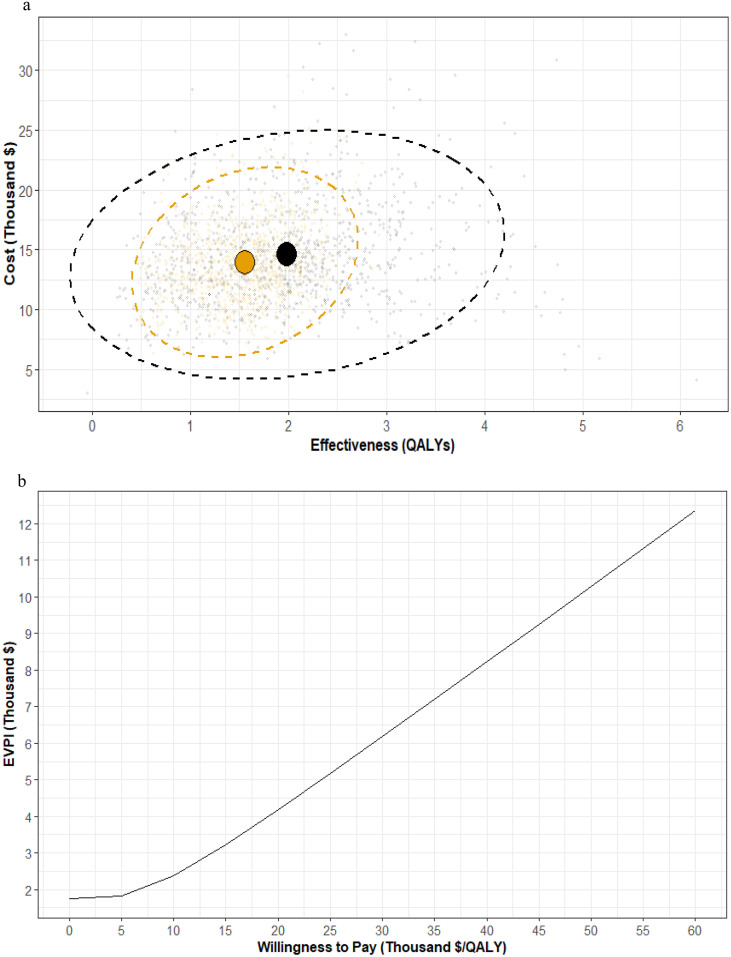
a: Cost-effectiveness scatter plot. b: Expected value of perfect information.

1) First step: we realized a Monte Carlos simulation that allows us to sample 1000 parameter sets.
2) Second step: we calculated the costs and QALYs of each strategy and produced the cost- effectiveness scatter plot [15], plotted as a point in the graph as well as the 95% confidence ellipse the (incremental cost, incremental QALYs) between both strategies (see Fig 3a).

According to the result, the Innovative strategy was more costly and more effective than SoC. As the willingness to pay (WTP) threshold is equal to €30,000 per QALY [16], the Innovative strategy has a probability of 85% of being more cost-effective than SoC, as determined by the Cost-effectiveness Acceptability Curve and a Net Monetary Benefit (NMB) of € 45375.74. At the same WTP threshold of €30,000 per QALY, the EVPI is €6,200 (Fig 3b). EVPI allows to assess the need for a new study to reduce uncertainty surrounding the decision-making process. The uncertainty surrounding the effectiveness of the innovative therapy is very low (€6,200). Therefore, conducting a new study to reduce uncertainty around the decision-making process would be more expensive.

VII Discussion

In this article, we have shown how to implement a time-dependent breast cancer model and how to carry out a cost effectiveness analysis with a mathematical description in R. In this tutorial, we used two types of methods to introduce time dependence:

First, implementing time-dependency into the Markov transition probabilities by letting the model employ probabilities that vary according to time spent by the cohort. To carry out simulation-time dependence, we developed a 3-dimensional transition probability array to take into account the exact period when the simulation started. There are other methods to conduct a simulation-time dependence but they are difficult to realize because the number of operations increases at every cycle, potentially slowing down the simulation of the Cancer model [7].

Second, relaxing the Markov assumption so as to ‘build memory’ into the model by using tunnel states. The patient moves through these tunnel states with transition probabilities, costs, or utility score that change over time. We noticed that time-dependent structure or tunnel states improve the result than simple Markov model because they bring more precision on patient health state evolution. There are important differences in estimated ICERs, and decision conclusions. With the R language, we can add reasonably complex tunnel states but that requires a computer with RAM large enough to store the transition probability arrays and results. Another approach is to combine the two methods, simulation-time and state- residence [17]. However, this could necessitate a 4-dimensional array, which may prove challenging.

For these reasons, it may be preferable to use microsimulation models instead of a cohort model [18]. In a microsimulation model, the risks and rewards of simulated individuals do not need to depend only on the current health state as they can also take into account their characteristics and attributes. We refer the reader to other literature on this subject [19–22].

To introduce time dependence in the breast cancer model, we used the R programming language, but almost the same code structure could be used in many other programming languages, such as MATLAB and Python. However, to improve the transparency and maximize the flexibility of decision-making models or health economic evaluation (EE) in R, several packages have been written, such as Markov Models for Health Economic Evaluations (heemod) [23], Health Economic Simulation Modeling and Decision Analysis (hesim) [24], and Bayesian Cost-Effectiveness Analysis (BCEA) [25]. Those R packages are developed to support modeling of the cohort or the treatment strategies and favour a reduction of programming time, because the programming has already been realized. The hesim package is more effective than other loops in R, because all the memory preallocation and loops are made at the C++ level, but that requires more RAM.

The increasing number of R packages can be considered in light of an open source modeling movement in the health economics community [26]. Thus, these packages, offering a variety of possibilities, make it easier and more robust to implement and analyse a wide variety of models using R [26].

## Conclusion

The current trend is to use software applications that can improve decision-making models and that can resolve more complex problems, as well as ensure the reproducibility and transparency of studies. The intention of this tutorial paper was to provide R codes with their explanation to help the beginning modeller and MS Excel user to switch to R without having any great knowledge of programming with R. All code for the R model used in this tutorial can be found at https://github.com/KKJM1/MODELING-IN-R-ADVANCED-METHOD-/tree/main.
